# Pharmacists’ Perceptions of 3D Printing and Bioprinting as Part of Personalized Pharmacy: A Cross-Sectional Pilot Study in Bulgaria

**DOI:** 10.3390/pharmacy13030088

**Published:** 2025-06-19

**Authors:** Anna Mihaylova, Antoniya Yaneva, Dobromira Shopova, Petya Kasnakova, Stanislava Harizanova, Nikoleta Parahuleva, Rumyana Etova, Ekaterina Raykova, Mariya Semerdzhieva, Desislava Bakova

**Affiliations:** 1 Department of Health Care Management, Faculty of Public Health, Medical University of Plovdiv, 4002 Plovdiv, Bulgaria; petya.kasnakova@mu-plovdiv.bg (P.K.); ekaterina.raykova@mu-plovdiv.bg (E.R.); mariya.semerdzhieva@mu-plovdiv.bg (M.S.); desislava.bakova@mu-plovdiv.bg (D.B.); 2 Department of Medical Informatics, Biostatistics and e-Learning, Faculty of Public Health, Medical University of Plovdiv, 4002 Plovdiv, Bulgaria; antoniya.yaneva@mu-plovdiv.bg; 3 Department of Prosthetic Dentistry, Faculty of Dental Medicine, Medical University of Plovdiv, 4002 Plovdiv, Bulgaria; dent.shopova@gmail.com; 4 Department of Hygiene, Faculty of Public Health, Medical University of Plovdiv, 4002 Plovdiv, Bulgaria; stanislava.harizanova@mu-plovdiv.bg; 5 Department of Obstetrics and Gynaecology, Medical University of Plovdiv, 4002 Plovdiv, Bulgaria; nikoleta.parahuleva@mu-plovdiv.bg; 6 Department of Epidemiology and Disaster Medicine, Faculty of Public Health, Medical University of Plovdiv, 4002 Plovdiv, Bulgaria; rumyana.etova@mu-plovdiv.bg

**Keywords:** 3D printing, bioprinting, pharmacists, personalized medicine, personalized pharmacy

## Abstract

Advances in pharmaceutical technology have positioned 3D printing and bioprinting as promising tools for developing personalized drug therapies. These innovations may redefine compounding practices by enabling precise, patient-specific drug formulations. Evaluating pharmacists’ readiness to adopt such technologies is therefore becoming increasingly important. **Aim:** The aim of this study is to investigate pharmacists’ knowledge, attitudes, and perceived barriers regarding the application of 3D printing and bioprinting technologies, as well as their perspectives on the regulation and implementation of these technologies in the context of personalized pharmacy. **Materials and Methods:** A custom-designed questionnaire was developed for the purposes of this pilot study, based on a review of the existing literature and informed by expert consultation to ensure conceptual relevance and clarity. The survey was conducted between September and December 2024. The data collection instrument comprises three main sections: (1) sociodemographic and professional characteristics, (2) knowledge regarding the applications of 3D printing and bioprinting in pharmacy, and (3) attitudes toward the regulatory framework and implementation of these technologies. **Results:** A total of 353 respondents participated, and 65.5% of them (n = 231) correctly distinguished between the concepts of “3D printing” and “bioprinting.” More than 25% (n = 88) were uncertain, and 8.5% (n = 30) were unable to differentiate between the two. Regarding the perceived benefits of personalized pharmacy, 83% (n = 293) of participants identified “the creation of personalized medications tailored to individual needs” as the main advantage, while 66% (n = 233) highlighted the “optimization of drug concentration to enhance therapeutic efficacy and minimize toxicity and adverse effects.” Approximately 60% (n = 210) of the pharmacists surveyed believed that the introduction of 3D-bioprinted pharmaceuticals would have a positive impact on the on-site preparation of customized drug formulations in community and hospital pharmacies. Lack of regulatory guidance and unresolved ethical concerns were identified as primary barriers. Notably, over 40% (n = 142) of respondents expressed concern that patients could be subjected to treatment approaches resembling “laboratory experimentation.” Nearly 90% (n = 317) of participants recognized the need for specialized training and expressed a willingness to engage in such educational initiatives. **Conclusions:** Three-dimensional printing and bioprinting technologies are considered cutting-edge instruments that may contribute to the advancement of pharmaceutical practice and industry, particularly in the field of personalized medicine. However, respondents’ views suggest that successful integration may require improved pharmacist awareness and targeted educational initiatives, along with the development and adaptation of appropriate regulatory frameworks to accommodate these novel technologies in drug design and compounding.

## 1. Introduction

In recent years, 3D printing and bioprinting have emerged as innovative technologies with the potential to transform pharmaceutical practice and industry [1,2]. These technologies offer novel opportunities for personalized medicine, optimization of drug formulations, and enhancement of therapeutic efficacy. They underscore the promise of developing advanced, individualized, and multifunctional platforms for drug delivery and testing [3,4,5,6].

### 1.1. 3D Printing and Bioprinting in Pharmaceutical Practice

Three-dimensional (3D) printing refers to the process of creating 3D structures of various shapes and sizes through the layer-by-layer deposition of materials, guided by computer-aided design (CAD) software. In the pharmaceutical domain, several 3D printing techniques are under investigation, including powder-based solidification (e.g., drop-on-solid, selective laser sintering), liquid-based solidification (e.g., stereolithography (SLA), microneedles, and drop-on-drop printing), and extrusion-based methods (e.g., fused deposition modeling (FDM) and room-temperature extrusion). Currently, the most widely applied techniques include powder solidification and extrusion-based printing [5,7].

Three-dimensional printing enables the fabrication of complex pharmaceutical dosage forms with precise control over drug dosage and release profiles, which is particularly advantageous for developing personalized therapies tailored to the individual needs of patients. It allows the production of multilayered drug systems designed for controlled release or custom-designed tablets for accurate dosing. Moreover, it can produce intricate structures that may enhance drug absorption or minimize adverse effects [8].

Spritam^®^, approved by the U.S. Food and Drug Administration (FDA) in 2015, is the first 3D-printed drug available on the market [9,10]. It is a highly porous tablet that disintegrates within seconds and is indicated for the treatment of seizures in both adults and children. This milestone has significantly advanced the concept of personalized drug delivery and has stimulated a surge in research initiatives focusing on the use of 3D printing for the customization of drug dosage, formulation, structure, and release characteristics [5].

Today, 3D printing technology is utilized throughout the drug development process, from preclinical research to clinical trials and frontline therapy. It is applied to various types of drug delivery systems, including oral controlled-release systems, microtablets, microchips, implants, microneedles, fast-dissolving tablets, and multiphase drug formulations. Compared to conventional pharmaceutical manufacturing methods, 3D printing offers numerous advantages, such as high production flexibility due to adaptable operating systems, and high drug loading capacity with desired precision and accuracy, particularly important for potent drugs administered at low doses [10].

Bioprinting, in contrast, employs living cells and biomaterials to create three-dimensional tissues and organs. It utilizes bioinks—composed of living cells and biomaterials—to construct organoids (tissue/organ equivalents) that replicate disease characteristics. In the pharmaceutical industry, bioprinting is increasingly used to develop models for drug discovery and testing, reducing reliance on animal models, improving the predictability of clinical outcomes, and enhancing the precision of drug testing.

The Organ-on-a-Chip (OoC) technology is emerging as a revolutionary alternative in drug development, aiming to replicate the environmental, functional, and interconnected characteristics of human organs and tissues [11,12]. This innovative approach provides enhanced insight into cellular mechanical properties, morphology, and differentiation, and facilitates the evaluation of cellular responses, gene expression profiles, and cellular functionality [13]. These systems exert a significant influence on the selection and evaluation of drug candidates with notable therapeutic potential, facilitating the design of novel therapeutics targeting specific mechanisms, and promoting therapeutic advances in hard-to-treat conditions, rare diseases, and regenerative medicine.

Major pharmaceutical companies are actively exploring 3D bioprinting as a tool for generating preclinical study models, highlighting its significance as a promising research approach within the pharmaceutical industry. This technology holds substantial potential for improving the efficiency of drug development, reducing costs, and shortening the time-to-market for new therapies [14,15]. It represents one of the most advanced and promising technologies in the field of personalized pharmacy.

### 1.2. Applications of 3D Printing in Personalized Pharmacy

Today, pharmacy is facing the challenge of adapting to personalized medicine. This paradigm can be referred to as personalized pharmacy—a revolutionary approach within the medical and pharmaceutical fields that aims to tailor pharmacotherapy to the individual needs of each patient. Rather than applying a one-size-fits-all strategy, personalized medicine leverages genetic information, biomarkers, and emerging technologies to provide more effective, targeted, and safer treatment options.

Traditional pharmacy relies on standardized dosage forms, fixed dosages, and treatment regimens guided by clinical protocols. However, individual differences in genetics, metabolism, age, sex, and lifestyle significantly influence both the efficacy and safety of medications. This is where 3D printing and bioprinting come into play as essential components of personalized pharmacy, contributing across several key domains:

- Pharmacogenomics, which explores how genetic variations affect individual responses to drugs;
- Biomarkers, measurable indicators that assist in selecting the most appropriate therapeutic strategies;
- 3D-printed pharmaceuticals, which allow for the development of individualized dosages and drug delivery forms tailored to specific patient profiles;
- Data analytics and artificial intelligence, used to predict the most effective therapeutic interventions for individual patients [16].

These advancements collectively support the shift toward precision pharmacotherapy, ultimately enhancing treatment outcomes and minimizing adverse effects through patient-specific drug design and delivery.

### 1.3. Pharmacists’ Role in Personalized Pharmacy

Pharmacists play a pivotal role in the implementation of personalized pharmacy. They are involved in counseling patients on genetic testing and potential drug interactions, preparing individualized dosages and pharmaceutical forms through 3D printing, and ensuring the safety and efficacy of drug combinations. Despite their importance, personalized medicine and pharmacogenetics are still regarded as emerging fields of patient care, and the roles and responsibilities of pharmacists in these areas remain not clearly defined [17].

Pharmacists are also key actors in integrating 3D printing into clinical practice. They can contribute to the development and manufacturing of personalized drug formulations, ensuring compliance with regulatory standards and maintaining the safety and effectiveness of the final products. Moreover, pharmacists can provide patient education on the use of 3D-printed medicines, including potential benefits and associated risks. Globally, there is a discernible trend shifting pharmacy services from generalized provision to more personalized, clinical, and specialized pharmaceutical care [7].

A study by Rautamo et al. on the application of 3D printing in the pharmaceutical sector reveals positive attitudes among healthcare professionals toward its adoption. Participants recognized several advantages, such as accurate dosing and the potential for individualized dosage forms. This was particularly relevant in cases of polypharmacy, where multiple active pharmaceutical ingredients could be combined into a single dosage form—known as a polypill—customized in both composition and dose. Such formulations could improve therapeutic outcomes and patient adherence in the treatment of various medical conditions [18]. In hospital settings, 3D and bioprinting technologies offer new opportunities for pharmacists engaged in extemporaneous compounding, allowing for the on-demand preparation of individualized dosage forms tailored to specific patient needs, particularly in pediatrics, oncology, and rare disease management [19,20].

### 1.4. Barriers to Implementation and Training Needs. Regulatory Challenges

Despite these benefits, 3D printing and personalized pharmacy face several challenges, including the urgent need for targeted education and training of pharmacists and physicians to enable the effective application of personalized therapies. Research shows that pharmacists express interest in adopting 3D printing within their practice, but significant obstacles remain, primarily related to insufficient knowledge and lack of formal training in this area. The need for specialized training programs is critical to familiarize pharmacists with the technological aspects and regulatory requirements related to pharmaceutical 3D printing [7].

Pharmacists and other healthcare professionals must keep pace with the rapid advancement of drug technologies. As high-tech medicines become more common in pharmaceutical practice, pharmacists are expected to continually update their knowledge of advanced drug technologies and assume a leading role in guiding society through this transformation [21].

Regulatory barriers requiring legislative adaptation represent a significant hindrance to the advancement of 3D printing in pharmacy. As of today, there are no specific regulations, recommendations, or guidelines governing the use of 3D printing in pharmaceutical products, despite numerous scientific publications and patent applications relating to its medical and pharmaceutical applications.

The U.S. Food and Drug Administration (FDA) issued the guideline “*Technical Considerations for Additive Manufactured Medical Devices*” in 2017 [22], which outlines recommendations for 3D-printed medical devices, covering aspects from device development to process validation and post-production activities. This guidance discusses a wide range of considerations in the design and manufacture of medical devices, including requirements for testing and labeling. However, the FDA has not yet issued any guidelines or regulations specifically regarding the use of 3D printing in drug manufacturing.

The European Medicines Agency (EMA) of the European Union also plays a crucial role in developing standards and guidelines for this emerging field [23], yet to date, it has not released specific regulations for 3D-printed pharmaceutical dosage forms. Manufacturers of 3D printers used for producing medical devices and pharmaceuticals are required to conduct risk assessments in accordance with the Machinery Directive 2006/42/EC, to determine applicable health and safety requirements for such equipment [24]. Nevertheless, the EU legal framework is technology-neutral, which means it does not impose specific technical solutions for product design. As a result, manufacturers are free to employ diverse technical approaches to meet the essential requirements [10]. While Bulgaria currently lacks specific regulatory guidance for 3D-printed pharmaceuticals, insights from FDA and EMA initiatives may serve as valuable models for shaping future national policies and aligning local pharmacy practice with emerging international standards.

While these technologies offer personalization, improved planning, and novel drug delivery systems, they also raise quality control and regulatory challenges, including material selection, process validation, sterilization, and scalability. Therefore, regulatory bodies must update their guidelines to ensure continued safety and efficacy. Looking ahead, with the integration of artificial intelligence, nanotechnology, and four-dimensional (4D) printing, future advancements may yield highly complex medical devices and revolutionize disease management and outcomes. Although 3D printing opens new avenues for pharmaceutical innovation, concerns remain regarding its scalability and regulatory compliance. As such, this technology is expected to have a profound impact on healthcare and pharmaceutical services in the coming decades, reshaping both the global research and regulatory landscapes [25].

The aim of this study is to investigate pharmacists’ and pharmacy assistants’ knowledge, attitudes, and perceived barriers regarding the application of 3D and bioprinting, as well as their views on regulation and the implementation of these technologies in personalized pharmacy.

## 2. Materials and Methods

### 2.1. Study Design and Participants

A cross-sectional survey was conducted between September 2024 and December 2024. The questionnaire was distributed electronically via professional pharmacy networks, institutional mailing lists, and social media platforms targeting licensed pharmacists.

### 2.2. Questionnaire Development and Structure

A self-administered online questionnaire was specifically developed for this study. It comprised 33 items, organized into three sections. The questionnaire was developed for the purposes of this pilot study. Content validity was assessed by experts in pharmacy practice and pharmaceutical sciences. The first section collected sociodemographic and professional characteristics, including age, sex, profession, and years of working experience. The second section assessed pharmacists’ knowledge of 3D printing and bioprinting. Respondents were asked to self-rate their level of awareness regarding these technologies using predefined options: “Fully aware”, “Partially aware”, or “Not aware”. The third section included questions covering attitudes and perceptions on regulatory concerns, ethical concerns, and financial motivation, as well as training needs. Most items assessing attitudes and perceptions were formatted using a 3-point scale with the following response options: Agree, Disagree, and Cannot decide.

### 2.3. Data Collection

The survey was anonymous and administered through Google Forms. Participants self-confirmed eligibility before beginning the questionnaire. Due to the anonymity of the format, duplicate submissions could not be fully controlled, but this approach was selected to encourage openness and increase the response rate. No follow-up reminders were issued to increase participation.

Prior to accessing the questionnaire, participants were shown an introductory statement outlining the study’s purpose, confidentiality assurance, and voluntary participation. Informed consent was implied by the participant’s decision to proceed and complete the survey. Following consent, participants completed the questionnaire via an online platform. No personal identification information was collected. Responses were automatically recorded and exported in CSV format.

### 2.4. Ethical Approval

The study protocol received approval from the Institutional Review Board of the Medical University of Plovdiv (Protocol R-2503/11.11.2022).

### 2.5. Statistical Analysis

Descriptive statistics were calculated for all variables: means ± standard deviations for continuous variables (e.g., age, years of experience) and as absolute frequencies and percentages for categorical measures. Pearson’s chi-square test was applied to examine associations between categorical variables. Assumptions for the test were met, including independence of observations and expected cell frequencies ≥ 5 in at least 80% of cases. All tests were two-tailed, and statistical significance was set at *p* < 0.05. Data analysis was conducted using IBM SPSS Statistics version 23. Tables and figures were generated in Microsoft Excel 2021.

## 3. Results

### 3.1. Demographic Characteristics

The study included pharmacists with a wide range of ages and experience in pharmaceutical practice. A total of 353 respondents participated, with a mean age of 38.37 ± 10.58 years, of whom 243 (68.8%) were women and 110 (31.2%) were men. Sociodemographic characteristics are summarized in Table 1.

### 3.2. Awareness and Knowledge of 3D (Bio)Printing

Pharmacists demonstrated partial awareness regarding the application of 3D printing (69.7%) and 3D bioprinting (67.4%) in the pharmaceutical sector. Only 12.5% of participants reported being fully informed about the use of 3D printing for drug development purposes, and 10% stated they were fully informed about the application of 3D bioprinting for drug testing.

A statistically significant gender difference in awareness levels was found. Women demonstrated higher awareness both in terms of the use of 3D printing in drug development (χ^2^ (1, N = 353) = 26.59, *p* = 0.001) and the use of 3D bioprinting in drug testing (χ^2^ (1, N = 353) = 21.05, *p* = 0.001).

Respondents primarily obtained information about innovative technologies from the internet, with over 68% using specialized medical websites and more than 46% relying on social media. Approximately half of the pharmacists reported obtaining information from scientific forums, conferences, and seminars. Just over 37% gained their knowledge during their pharmacy education, and 25.5% through postgraduate courses and professional qualifications covering innovative technologies.

Statistically significant gender-based differences were also identified in the sources of information. Men were more likely to rely on social media, χ^2^ (1, N = 353) = 7.92, *p* = 0.005, and postgraduate training, χ^2^ (1, N = 353) = 4.40, *p* = 0.010, while women more frequently reported attending scientific seminars, χ^2^ (1, N = 353) = 10.61, *p* = 0.001.

A statistically significant age-related difference was also found. Participants over the age of 46 were the most informed regarding the application of 3D printing in drug development, χ^2^ (6, N = 353) = 84.90, *p* = 0.001. Respondents aged up to 25 most commonly reported medical websites, χ^2^ (5, N = 353) = 11.01, *p* = 0.012, social media χ^2^ (5, N = 353) = 11.45, *p* = 0.009, and pharmacy education, χ^2^ (5, N = 353) = 26.05, *p* = 0.001, as key sources of information. Respondents aged 26–35 preferred most frequently obtained information from conferences, χ^2^ (5, N = 353) = 9.15, *p* = 0.027, whereas the 36–45 age group most often referred to postgraduate training, χ^2^ (5, N = 353) = 63.91, *p* = 0.001.

A similar understanding of the terms “*3D printing*” and “*bioprinting*” was most frequently observed among participants under 25 and over 46, who did not perceive substantial differences between the two terms, χ^2^ (6, N = 353) = 42.21, *p* = 0.001.

Regarding the definition of 3D printing, 22.1% of respondents selected “*the creation of dosage forms using biocompatible materials*”, while 65.4% chose “*three-dimensional prototyping using computer modeling and layer-by-layer fabrication of drug dosage forms*”. For 3D bioprinting, 42.5% selected the full definition, “*the creation of spatial structures from biocompatible materials populated with living cells*”, whereas 39.1% limited it to “*the creation of spatial structures from biocompatible materials*”. Only 11.6% defined it solely as “*the creation of spatial structures from living cells*”.

**Figure 1:**
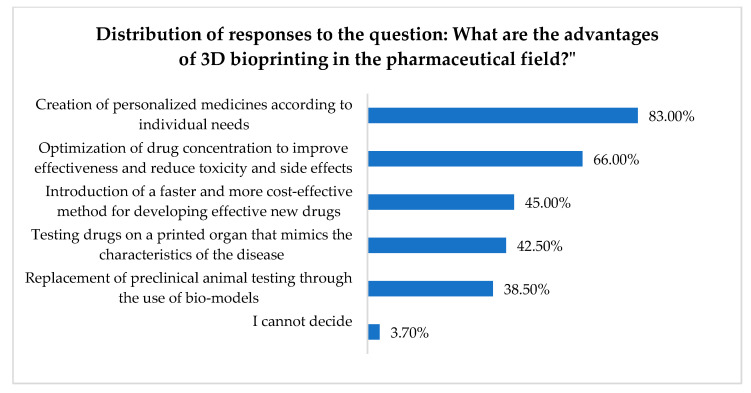
Some advantages of 3D (bio)printing.

Figure 1 presents the distribution of responses regarding some perceived advantages of 3D (bio)printing in the pharmaceutical field.

Over 73% of pharmacists consider personalized pharmacy as the most applicable field, with the creation of personalized medicines for individual therapy being the most common choice among the largest group of participants. The development of specific drugs to control the release rate and tissue-specific efficacy in drug delivery is the next most frequent choice, supported by 35–45% of pharmacists. The results showed that 35% of participants believe that 3D printing of personalized pharmaceutical forms can be implemented in pharmaceutical manufacturing activities in pharmacy practice, alongside the manual preparation of extemporaneous drug formulations.

Pharmacists’ attitudes toward personalized pharmacy are also reflected in their responses when presented with the advantages of 3D (bio)printing in the pharmaceutical sector, as 83% of respondents identified “the creation of personalized medicines according to individual needs” as the primary advantage of this technology. “Optimizing drug concentration to improve efficacy and reduce toxicity and side effects” was identified as an advantage by 66% of participants.

Reducing the time and cost of clinical trials and the market introduction of new, effective drugs is considered a major advantage by 45% of respondents. “Testing drugs on a printed organ mimicking disease characteristics” and “replacing preclinical animal testing with bio-models” were identified as advantages of 3D (bio)printing by 38% and 42% of respondents, respectively.

While specialists up to 25 years old more frequently believe that the primary advantage of 3D bioprinting in the pharmaceutical field is “creating personalized medicines according to individual needs”, χ^2^ (5, N = 353) = 18.75, *p* = 0.001, those aged 36–45 years tend to view the advantage as “introducing a faster and cheaper method for creating effective new drugs” χ^2^ (5, N = 353) = 10.41, *p* = 0.015. Participants aged 26–35 more often believe that the advantages of 3D (bio)printing in the pharmaceutical field are related to optimizing drug concentration for improving efficacy and reducing toxicity and side effects (χ^2^ (3, N = 353) = 59.10, *p* < 0.001), testing drugs on a printed organ mimicking disease characteristics (χ^2^ (3, N = 353) = 13.05, *p* = 0.005), and replacing preclinical animal testing with bio-models (χ^2^ (3, N = 353) = 13.78, *p* = 0.003).

### 3.3. Attitudes and Perceptions of Personalized Medicine and Personalized Pharmacy

Participants’ perceptions regarding the significance of personalized medicine/pharmacy were evaluated. The ability of 3D printing to produce accurate doses and personalized drug release on a small scale makes it a promising method for manufacturing personalized treatment based on an individual’s health profile. A positive perception was observed in the participants’ responses regarding the importance of personalized medicine. The majority of participants, 74%, believe that personalized medicine will help overcome differences in the genetic and metabolic profiles of patients, while 17.3% provided neutral responses, and 8.8% responded negatively. A total of 248 participants (70.3%) agreed that the patient’s genetic profile could influence their response to personalized drug therapy, while 67 participants (19%) were unsure, and 38 participants (10.8%) disagreed. Approximately three-quarters of participants agreed with the following statements: “the safety and efficacy of drug therapy can be improved through the use of 3D (bio)printing in the preparation of personalized medicines” and “3D (bio)printing will allow the preparation of personalized medicines for all age groups.” A positive attitude was expressed regarding “personalizing the dosage and form of administration will improve patient adherence to treatment” and “3D (bio)printing will support the production of medicines for rare diseases and reduce drug waste,” with respective agreements of 77.9% and 73.9%.

For the purposes of our study, it was important to determine the level of agreement among respondents regarding certain statements (Table 2).

Young specialists up to 25 years old most frequently believe that 3D bioprinting can be used in the creation of models suitable for testing and identifying new pharmaceutical molecules, χ^2^ (5, N = 353) = 52.43, *p* < 0.001, the creation of specific drugs to control the release rate, χ^2^ (5, N = 353) = 112.69, *p* < 0.001, and the creation of drugs for “individualized therapy” based on the biological and clinical parameters of the patient (χ^2^ (5, N = 353) = 15.22, *p* = 0.002). Meanwhile, participants aged 26–35 more frequently believe that 3D bioprinting can be used in drug delivery and tissue-specific efficacy (χ^2^ (5, N = 353) = 22.69, *p* = 0.001), in vitro predictive toxicology (χ^2^ (5, N = 353) = 51.49, *p* = 0.001), high-throughput screening (χ^2^ (5, N = 353) = 32.83, *p* = 0.001), and drug testing on printed human cell models (χ^2^ (5, N = 353) = 39.99, *p* = 0.001).

The application of 3D printing in pharmaceutical manufacturing activities in the pharmacy is most highly rated by the group under 25 years old, (χ^2^ (5, N = 353) = 18.52, *p* = 0.003).

### 3.4. Disadvantages of 3D (Bio)Printing

The survey results show differences in perceptions regarding the drawbacks of 3D bioprinting across different age groups, as well as regarding the potential risks and limitations of the technology. The group aged 36–45 considers the main drawback to be the “introduction of live cells” (χ^2^ (5, N = 353) = 55.09, *p* = 0.001). Those under 25 years old more frequently consider the risk of “contamination with potential endogenous viruses when using animal biological material” (χ^2^ (5, N = 353) = 14.22, *p* = 0.003). Both those under 25 and over 46 years old more frequently believe that there is an “uncontrolled desire for self-improvement of the physical body” (χ^2^ (5, N = 353) = 20.07, *p* = 0.001). Participants under 25 years old identify 3D bioprinting as an “insufficiently researched technology with unknown side effects” (χ^2^ (5, N = 353) = 85.61, *p* = 0.001).

### 3.5. Attitudes Towards Regulation and Implementation of 3D Bioprinting in the Pharmaceutical Sector

The topic of regulation and implementation of 3D bioprinting in the pharmaceutical sector provokes various attitudes among pharmacists. Key aspects influencing their views include the need for strict control and regulation by specialized institutions regarding the application of the technology, as well as the preference for research centers (90.1%), hospitals, and healthcare facilities (approximately 63%) to be the primary entities working with these technologies. Fewer than 50% of respondents indicated pharmaceutical organizations (Table 3).

More than half of the respondents believe that 3D bioprinting should be applied when conventional medical methods have been exhausted, despite the potential risk of irreversible consequences for the patient. However, the lack of regulation and emerging ethical issues are concerns for many respondents, with over 40% believing that the patient could become an “experimental subject”.

Nevertheless, pharmacists believe that the introduction of 3D-bioprinted medicines would have a positive impact on pharmaceutical manufacturing activities in pharmacies (nearly 60%). However, they declare the need for specialized training, with nearly 90% expressing a positive attitude towards participating in additional training and courses to enhance their knowledge in the field of 3D bioprinting. About 70% of pharmacists prefer the training to be interactive and conducted in the form of seminars and live demonstrations (Table 4).

The survey results show differences in opinions regarding the right to use (bio)printers by different institutions and organizations, depending on the respondents’ experience. Participants with more than 21 years of experience more frequently respond that healthcare organizations, pharmaceutical companies, and medical and dental centers should not have the right to use this technology (χ^2^ (6, N = 353) = 26.87, *p* = 0.001; χ^2^ (6, N = 353) = 36.84, *p* = 0.001; χ^2^ (6, N = 353) = 11.46, *p* = 0.022). At the same time, participants with up to 5 years of experience believe that outpatient specialized medical centers should have the right to use (bio)printers (χ^2^ (6, N = 353) = 60.51, *p* = 0.001).

Regarding the question of whether (bio)printing should be applied when conventional medical methods have been exhausted, despite the risk of irreversible consequences for the patient (Table 2), participants with 16–20 years of experience more frequently respond positively (χ^2^ (6, N = 353) = 88.79, *p* = 0.001) compared to other age groups.

## 4. Discussion

The findings of our study show a partially comparable trend with those reported in the Saudi Arabian study. While 53% of Saudi pharmacists reported general familiarity with 3D printing, a higher percentage of Bulgarian pharmacists (69.7%) indicated partial awareness of its pharmaceutical applications [26]. Similarly, both studies found low levels of full awareness: 12.5% in our study versus 14–16% in the Saudi study, suggesting that detailed knowledge about clinical use remains limited across contexts. Despite limited knowledge, 67% of pharmacists express willingness to adopt this new technology in their practice, reflecting their openness to innovation. The main barriers to implementing 3D printing are associated with the cost of the technology, regulations, and the shortage of practicing pharmacists [26,27].

A study conducted among pharmacy students at the University of Copenhagen shows that 43 out of 51 respondents believe 3D printing can play a role in the development of personalized medicine. Thirty-one students believe that the implementation of 3D printing will directly affect pharmaceutical services, and thirty-five see it as an opportunity to provide better personalized treatment for patients. Most students consider the design of 3D tablets to be a collaborative effort between the doctor, pharmacist, and patient [28]. The positive attitudes of pharmacists and pharmacy students towards personalized medicine and 3D printing support the results of our study, in which two-thirds of the participants also have a positive attitude. They also highlight the need for additional training and the development of strategies to overcome barriers to implementing these technologies in clinical practice.

Pharmacists show significant interest in the integration of 3D bioprinting into the sector but express concerns about the regulatory challenges associated with this technology. Algahtani’s study indicates that 67% of pharmacists are inclined to adopt 3D printing as a method for personalized drug delivery, though they identify regulations as one of the main barriers to its implementation. The author of the study emphasizes the need for a strategic plan that includes collaboration between pharmacists, regulatory bodies, and 3D printing engineers to overcome these challenges. Despite the concerns, participants express a positive attitude toward the concept of personalized medicine via 3D printing, though they point to issues such as cost, regulations, and lack of experience as significant obstacles [26].

Moreover, another study highlights that while 3D printing offers significant opportunities for personalized medicine, there are still challenges regarding regulations and its practical application in pharmacy practice. The authors note the need to develop policies and guidelines to facilitate the integration of this technology into the sector [29]. These studies underline the importance of clear regulatory frameworks and guidelines that can facilitate the successful adoption of 3D bioprinting in the pharmaceutical sector.

Despite the progress and benefits for both the pharmaceutical field and patients, several challenges remain, such as the need for clear regulatory guidelines for integrating new manufacturing technologies in clinical settings, strategies to ensure the quality and safety of formulations produced in the clinic, and further engagement with healthcare professionals (including pharmacists), who will play a key role in the application and management of this technology in practice [30]. Well-thought-out regulatory decisions can accelerate the adoption of 3D printing in medicine to meet the primary needs of patients. Early successes are likely to be seen in highly personalized applications, particularly for challenging pediatric and geriatric dosing needs [31].

The main barriers to implementing 3D bioprinting in pharmaceutical practice are related to regulations, standardization, and the need for interdisciplinary collaboration between pharmacists, engineers, and regulatory bodies. The respondents of our study placed emphasis on several regulatory uncertainties surrounding the implementation of 3D printing and bioprinting technologies in pharmacy practice, including the absence of clear national or EU-level guidelines on the classification, quality standards, and legal status of 3D-printed medicinal products; uncertainty regarding liability in case of adverse events linked to pharmacy-produced 3D-printed formulations; and the lack of regulatory frameworks for pharmacist-led bioprinting. However, many professionals in the field are ready to adopt and implement these technologies if clear guidelines and adequate training are provided.

To facilitate the successful integration of 3D printing into the pharmaceutical sector, it is necessary to develop educational programs, improve regulatory frameworks, and encourage cooperation among key stakeholders. In the long run, these measures will support the adaptation of innovative technologies and improve the quality and personalization of treatment for patients.

## 5. Limitations

While this study provides valuable insights into pharmacists’ awareness and perceptions of 3D (bio)printing, several limitations should be noted. The cross-sectional design captures opinions at a specific point in time, which may not reflect evolving attitudes as technology matures. The questionnaire, although carefully developed and reviewed by experts in pharmacy practice, was not based on a previously validated tool. Additionally, self-reported data may be influenced by individual interpretation. Due to the anonymous nature of the survey, we were unable to verify participants’ professional licensure or prevent potential duplicate submissions, which may affect the generalizability of the findings.

## 6. Conclusions

Pharmacists in this study perceived 3D printing and bioprinting as promising technologies with potential applications in personalized drug therapy. Despite partial awareness, participants expressed a strong willingness to engage in specialized training, particularly through interactive formats.

These findings suggest that the integration of 3D (bio)printing into pharmacy practice may support more individualized approaches to drug preparation. However, effective implementation will depend on targeted educational initiatives and the development of appropriate regulatory frameworks.

The future of personalized pharmacy is promising, as with the development of technology, artificial intelligence, and bioengineering, drug therapy will become increasingly precise and effective.

## Figures and Tables

**Table 1:** Demographic characteristics (N = 353).

Characteristic	**n**	**%**
**Gender**		
*Women*	*243*	*68.8%*
*Men*	*110*	*31.2%*
**Age (years)**		
*Mean (SD)*	*38.37 (10.58)*	
**Age groups**		
*Up to 25*	*54*	*15.3%*
*26–35*	*88*	*24.9%*
*36–45*	*139*	*39.4%*
*Over 46*	*72*	*20.4%*
**Years of Experience**		
*Less than 4 years*	*76*	*21.5%*
*5–9 years*	*47*	*13.3%*
*10–14 years*	*81*	*22.9%*
*15–19 years*	*69*	*19.5%*
*More than 20 years*	*80*	*22.7%*
*Mean (SD)*	*12.46 (8.79)*	

**Table 2:** Statements related to the application of 3D (bio)printing.

Statement	I Agree n (%)	I Cannot Decide n (%)	I Disagree n (%)
The patient’s genetic profile can influence their response to personalized drug therapy.	248 (70.3%)	67 (19%)	38 (10.8%)
Personalized medicine will help overcome differences in the patient’s genetic and metabolic profile.	261 (73.9%)	31 (8.8%)	61 (17.3%)
Personalization of dosage and administration form will improve patient adherence to treatment.	275 (77.9%)	15 (4.2%)	63 (17.8%)
The safety and efficacy of drug therapy can be improved by using 3D (bio)printing in the preparation of personalized drugs.	295 (83.6%)	36 (10.2%)	22 (6.2%)
3D (bio)printing will allow the preparation of personalized drugs for all age groups.	293 (83%)	37 (10.5%)	23 (6.5%)
3D (bio)printing will support the production of drugs for rare diseases and reduce drug waste.	279 (79%)	15 (4.2%)	59 (16.7%)
3D (bio)printing has a place in the pharmaceutical manufacturing activity in the pharmacy (preparation of extemporaneous forms).	237 (67.1%)	56 (15.9%)	60 (17%)

**Table 3:** Regulation and implementation of 3D bioprinting in the pharmaceutical sector.

Statement	I Agree n (%)	I Cannot Decide n (%)	I Disagree n (%)
The application of 3D (bio)printing in personalized medicine should be exempt from legal regulation	36 (10.2%)	11 (3.1%)	306 (86.7%)
The possession and use of 3D (bio)printers should be regulated and controlled by an official institution	328 (92.9%)	5 (1.4%)	20 (5.7%)
Application of (bio)printing when conventional methods have been exhausted, despite the risk of irreversible consequences for the patient	180 (51%)	109 (30.9%)	64 (18.1%)
Potential risks would turn the patient into a “test guinea pig”	142 (40.2%)	98 (27.8%)	113 (32%)

**Table 4:** Distribution of responses regarding the authorization to use 3D (bio)printing, its impact on pharmaceutical manufacturing activities, and readiness for training among pharmacists (n, %).

Category	n (%)
Right to use (bio)printers	
Research centers	318 (90.1%)
Health organizations (hospitals, hospital pharmacies, etc.)	222 (62.9%)
Pharmaceutical companies and pharmacies	174 (49.3%)
Medical and dental centers	108 (30.6%)
Specialized medical care clinics	65 (18.4%)
Independent medical and technical laboratories	49 (13.9%)
Cannot assess	13 (3.7%)
Impact on pharmaceutical manufacturing activity	
Will have a negative effect	16 (4.5%)
Will have a positive effect	208 (58.9%)
Cannot assess	129 (36.5%)
Readiness for additional training/courses	
Yes	316 (89.5%)
No	22 (6.2%)
Cannot assess	15 (4.2%)
Preferred forms of training	
Live demonstrations	256 (72.5%)
Seminars	245 (69.4%)
Lectures	199 (56.4%)
Workshops	164 (46.5%)

## Data Availability

The original contributions presented in this study are included in the article. Further inquiries can be directed to the corresponding author.
